# Primary hepatic extranodal marginal zone B-cell lymphoma of mucosa-associated lymphoid tissue type: A case report and literature review

**DOI:** 10.1097/MD.0000000000006305

**Published:** 2017-03-31

**Authors:** Shuilin Dong, Lin Chen, Yifa Chen, Xiaoping Chen

**Affiliations:** Hepatic Surgery Center, Tongji Hospital, Tongji Medical College, Huazhong University of Science and Technology, Wuhan, China.

**Keywords:** liver hepatectomy, MALT lymphoma, marginal zone B-cell lymphoma, rituximab

## Abstract

**Rationale:**

Primary hepatic mucosa-associated lymphoid tissue (MALT) lymphoma is an extremely rare disease. To the best of our knowledge, only 67 cases had been reported in 39 English literatures to date. The aim of this study was to add a new case of this disease to the literature and to review the current literature.

**Patient concerns:**

A 50-year-old man was incidentally identified with a solitary mass of 5 cm in diameter in the left lobe of the liver.

**Diagnoses:**

Based on the results of imaging studies, intrahepatic cholangiocellular carcinoma was suspected, and then surgery was performed. Microscopic findings showed that the tumor was a hepatic MALT lymphoma, and immunohistochemical analysis revealed that the lymphoma cells were CD20+, CD79a+, BCL-2+, CD3−, and CD5−.

**Interventions:**

The patient received rituximab after surgery.

**Outcomes:**

He was free of disease for 13 months at the time of this report.

**Lessons:**

Since previously published case reports and our case described nonspecific clinical features of this rare disease, it was usually misdiagnosed before histological confirmation and surgery resection may be a good choice for both diagnosis and local therapy.

## 1 Introduction

Marginal zone B-cell malignant lymphoma refers to a low-grade malignant non-Hodgkin lymphoma that develops in mucosa-associated lymphoid tissue (MALT). Isaacson and Wright first proposed the concept of MALT lymphoma for extranodal malignant lymphoma of marginal zone B-cell origin in 1983.^[1]^ Subsequently, the World Health Organization and the Revised European American Lymphoma Classification categorized it as a distinct clinicopathological entity with characteristic histological features.^[2,3]^ MALT lymphoma is a relatively rare disease, nevertheless it represents the third most common type of lymphoma accounting for approximately 7% to 8% of all non-Hodgkin lymphomas. The stomach is the most commonly affected site; but MALT lymphomas can arise at any extranodal site, including the salivary gland, conjunctiva, thyroid gland, orbit, lung, larynx, breast, liver, skin, dura mater, etc.^[4,5]^

Primary hepatic MALT lymphoma is an extremely rare disease, and little is known about its clinical course and optimal treatment. To the best of our knowledge, only 67 cases had been reported in 39 English literatures to date, with 43 cases having detailed description of clinical presentations, pathologic features, therapy, and clinical outcome.^[5–43]^ Herein, we describe a case of surgically resected primary hepatic MALT lymphoma, which was initially suspected to be an intrahepatic cholangiocellular carcinoma. We also review the relevant literature to discuss the clinicopathological features and management of this extremely rare disease.

## 2 Case report

A 50-year-old man was referred to our hospital because of a solitary mass in the liver, which was identified incidentally by ultrasonography during an annual physical examination. On admission, the patient did not state any discomfort and the physical examination was unremarkable without palpable splenomegaly or superficial lymph node swellings. He underwent cholecystectomy 20 years ago and had a history of type 2 diabetes and hypertension for 10 years. The routine clinical laboratory parameters were as follows: Blood cell counts and serochemical findings including liver enzymes were normal. No hepatitis B virus, hepatitis C virus, or human immunodeficiency virus infection was found. Tumor biomarkers, including alpha fetoprotein (AFP), carbohydrate antigen 19-9 (CA19-9), and carcinoembryonic antigen (CEA), were unremarkable. In addition, autoimmune hepatitis tests were negative.

**Figure 1:**
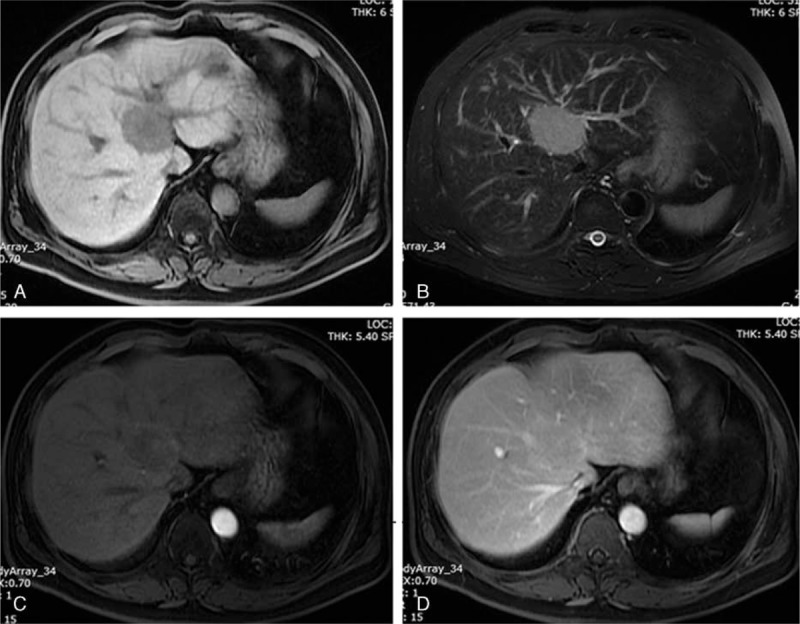
Enhanced magnetic resonance imaging findings. The mass was demonstrated low signal intensity on T1-weighted imaging (A) and slightly high signal intensity on T2-weighted imaging (B). The mass was slightly and peripherally enhanced in the arterial phase (C) and portal phase (D).

Abdominal ultrasound examination showed a 5.3 × 4.5 cm hypoechoic homogeneous mass in the segment IV (S4) adjacent to the hepatic portal. Enhanced magnetic resonance imaging (MRI) demonstrated a T1 hypointensity and T2 hyperintensity round mass located in the S4 of the liver, which was adjacent to the left portal vein branch and partially surrounding the bile duct of the left hepatic lobe. The mass was slightly and peripherally enhanced in the arterial phase and portal phase (Fig. 1). There were no extrahepatic mass shown by the MRI.

**Figure 2:**
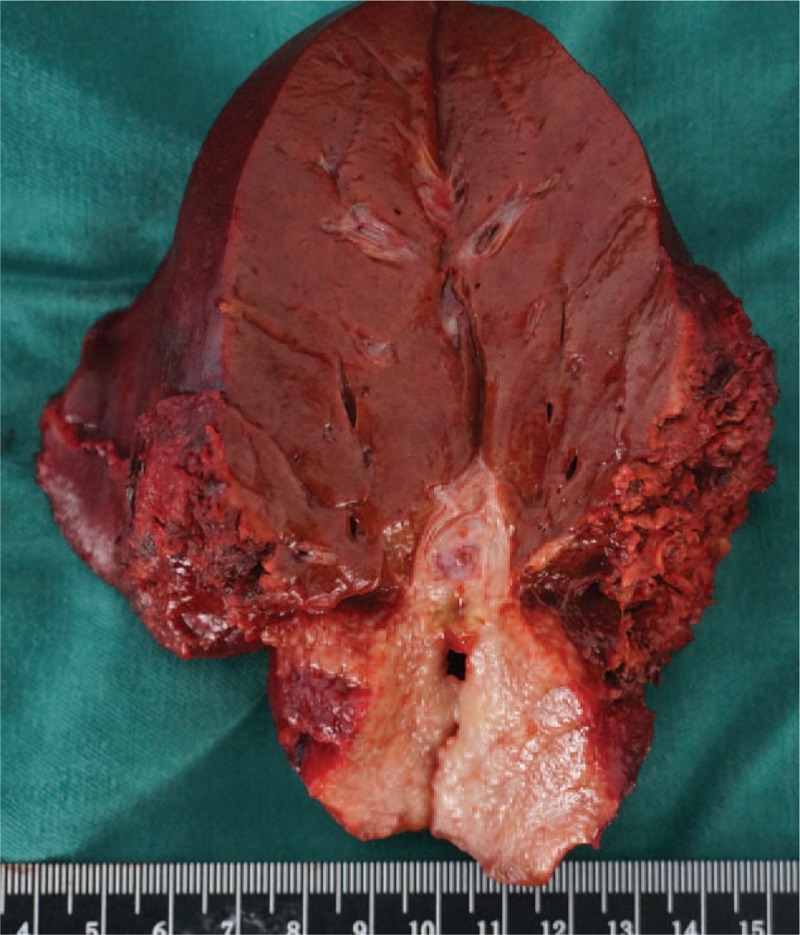
Gross appearance showed the cut surface of the tumor with a yellow-whitish firm pattern.

**Figure 3:**
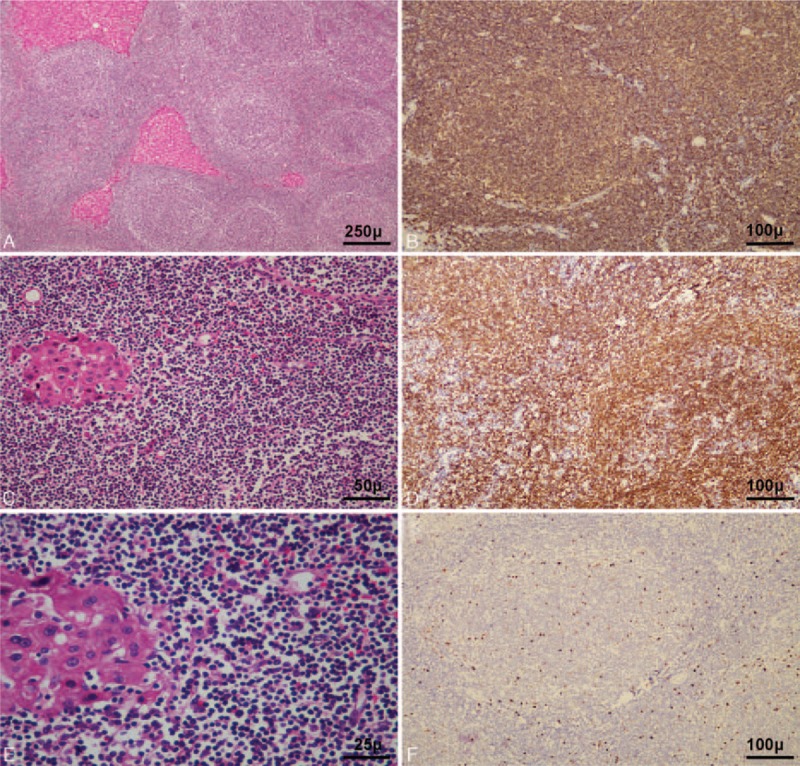
Characteristics of histological examination. The lesion consisted of dense lymphocyte infiltration with some lymphoid follicles (A, Hematoxylin and Eosin, HE ×40). Small- to middle-sized, centrocyte-like lymphoid cell population were diffusely infiltrated with lymphoepithelial lesions of bile ducts (B, HE ×200 and C, HE ×400). And lymphocytes were diffusely positive for CD20 (D) and CD79a (E) antibodies. Ki67 labeling index was in a low level (F).

According to the results of the imaging examinations, we suspected the tumor as an intrahepatic cholangiocellular carcinoma. Liver biopsy was not performed due to the risk of potential needle metastasis. The patient underwent left hemihepatectomy in December 2015. Macroscopically, the cut surface of the tumor showed a yellow-whitish firm pattern and the tumor was adjacent to the left portal tract (Fig. 2). Microscopic examination showed diffusion and monotonous infiltration of typical centrocyte-like lymphoid cells, and lymphoepithelial lesions could be observed on some bile ducts infiltrated with these small- to medium-sized lymphocytes. Immunohistochemistry findings were positive for CD20, CD79a, and BCL-2, whereas they were negative for CD3, CD5, CD10, CD43, and Cyclin D1. The Ki67 labeling index was in a low level (Fig. 3). As a result, the patient was diagnosed with low-grade hepatic MALT lymphoma based on the above-mentioned pathological findings by 2 experienced pathologists independently.

The patient's postoperative course was uneventful. Staging evaluations including an FDG-PET/computed tomography (CT) scan, upper gastrointestinal endoscope, and bone marrow aspiration showed no lesions other than in the liver. Upper endoscopic findings indicated chronic esophagitis, chronic erosive gastritis, and duodenal bulbar ulcer. But the ^13^C-urea breath test used to detect *Helicobacter pylori* infection was negative. According to the Ann Arbor staging, it was classified as a stage IE tumor.^[44]^ The patient received 6 courses of rituximab (375 mg/m^2^) monthly after resection in the Department of Hematology without obvious adverse effects. He remains well with no evidence of relapse for the following 13 months.

## 3 Discussion

MALT lymphomas are a subgroup of low-grade B-cell lymphomas that arise from extranodal sites normally devoid of lymphoid tissue. It is characterized by an indolent natural history and has a tendency to stay localized for a long time.^[45]^ Treatment with antibiotics and acid-reducing medications has become the standard first-line therapy for *H pylori*-associated gastric MALT lymphoma.^[46]^ However, there is no consensus on the optimal treatment for patients with nongastric MALT lymphoma. Surgery, radiotherapy, chemotherapy, or combinations have all been used with favorable results.^[5]^ Therein, primary hepatic MALT lymphoma is extremely rare, and little is known about its clinical features and optimal treatment. We only found 67 cases reported in 39 English literatures to date in PubMed and Medline database, with 43 cases having detailed descriptions of clinical presentations, pathologic features, therapy, and clinical outcomes. Almost all the patients lacked typical clinical features and could not be diagnosed definitely without histological results. Many cases had been misdiagnosed as other malignant tumors before biopsy or surgery. In this report, we described a case of incidentally finding primary hepatic MALT lymphoma, which was regarded as intrahepatic cholangiocellular carcinoma preoperatively. Consequently, the patient was treated with surgical resection and rituximab, and he remains free of disease for 13 months to the date of writing.

For the literature review, we summarized the clinical features of the reported cases in Table 1. There were 18 females and 29 males, with an age ranging from 36 to 85 years and a median age of 62 years. Total 68 patients (including the present case) consisted of 35 Europeans, 27 East Asians, and 6 Americans. Among the 27 East Asian patients, 21 were Japanese, 4 were Chinese, and 2 were Koreans. Most patients (85.4%) were asymptomatic and their liver lesions were incidentally found during examination or surgical explorations. The majority of patients (79.6%) presented with a solitary mass. The masses ranged from 0.7 to 9.0 cm in diameter. According to the Ann Arbor staging, 88.9% patients belonged to stage IE.

**Table 1:**
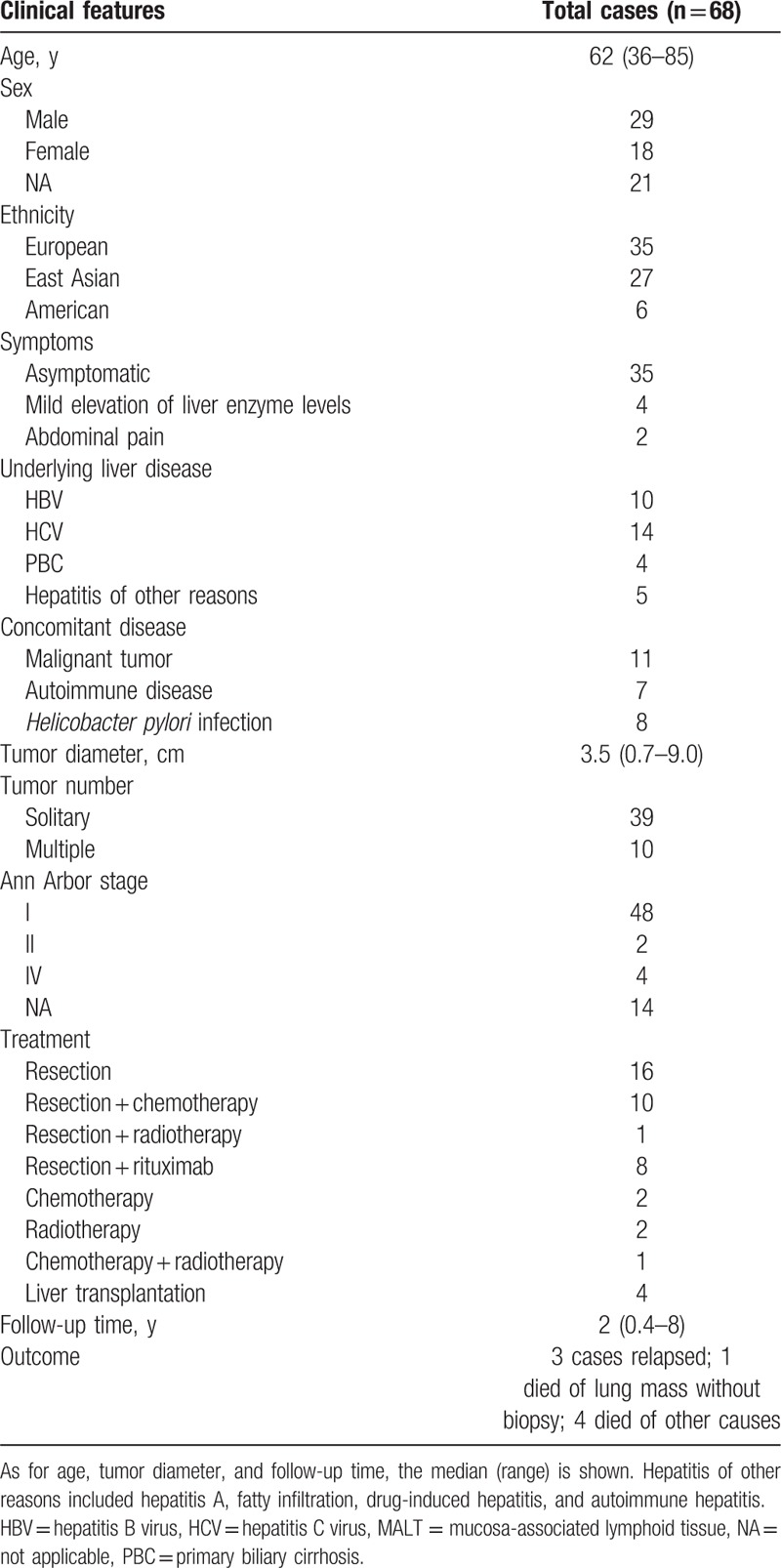
Clinical features of the patients with primary hepatic MALT lymphoma ever reported (including the present case).

MALT lymphoma usually arises in background of chronic inflammation associated with infective agents, such as *H pylori*-associated chronic gastritis,^[47]^ or autoimmune diseases, such Sjogren syndrome^[48]^ and Hashimoto thyroiditis.^[49]^ However, the etiology of hepatic malignant lymphomas, especially MALT lymphomas, remains unknown. Most hepatic MALT lymphoma cases suffered basic liver diseases, including primary biliary cirrhosis (4 cases), hepatitis C virus (14 cases), hepatitis B virus (10 cases), or hepatitis of other reasons (6 cases). In addition, 8 cases had *H pylori* infection as some authors had described that there was a strong association between the presence of *H pylori* in the stomach and in the bile juice.^[30,50]^ Moreover, 7 cases had autoimmune diseases and 11 cases had synchronous malignant tumors. These suggested that chronic inflammation might also contribute to development of the primary hepatic MALT lymphoma. However, more evidence is still required. As in our case, the patient's medical history was unremarkable without preceding liver diseases or *H pylori* infection.

As far as reported by the literatures, primary hepatic MALT lymphoma lacked specific clinical presentations and biomarkers. The serum levels of tumor markers, including AFP, CA19-9, and CEA, were all in normal range, except for 2 cases of liver cirrhosis with slightly evaluated AFP level.^[22,24]^ Meanwhile, distinctive radiological features were absent. Ultrasound studies showed majority of the hepatic MALT lymphomas as hypoechoic masses,^[7,15,16,22,26,29,31,40,43]^ which was in agreement with a previous study of liver lymphomas.^[51]^ On contrast-enhanced CT, primary hepatic MALT lymphoma had been described as low-density mass, which was not enhanced,^[16,17,31,39]^ or faint enhanced,^[15,36,40]^ or enhanced peripherally in the early arterial phase.^[29]^ MRI was characterized by low signal intensity on T1-weighted images and moderately high signal intensity on T2-weighted images, and the enhancement pattern was similar to that of CT.^[15,22,35,36,40,43]^ Our case also had these radiological features. Accordingly, the imaging features were similar to that of hepatocellular carcinoma, intrahepatic cholangiocellular carcinoma, or metastatic tumor, so the diagnosis was commonly misjudged. In most cases, the diagnosis of MALT lymphoma would not be first considered before histological confirmation. Nevertheless, radiological investigation is important to exclude other primary sites of MALT lymphoma.

Currently, there are no standard therapeutic protocols or guidelines for the treatment of primary hepatic MALT lymphoma. Surgery, chemotherapy, or radiotherapy alone, or in combination had been commonly used. Most patients received surgical resection among the reported cases (Table 1). Most patients were reported in good results with a median follow-up time of 2 years. Rituximab is an anti-CD20 monoclonal antibody that has been shown to be effective in MALT lymphoma with remission rates of 55% to 73% and no intolerable adverse effects.^[52,53]^ In a previous case report, a patient with hepatic MALT lymphoma which relapsed 14 months after resection achieved complete remission again after using rituximab alone.^[23]^ Because extragastric MALT lymphoma was reported to have a higher recurrence rate than gastric MALT lymphoma,^[54]^ we gave the patient rituximab after resection and he remains well without the disease to date. However, long-time follow-up is still expected for establishment of the best therapeutic methods for this disease, and additional accumulation of cases is needed to establish effective diagnostic methods.

## 4 Conclusion

In the present report, we described a case of primary hepatic MALT lymphoma. Our experience in this case and review of relevant literature indicated primary hepatic MALT was rare with incidental finding. It has a tendency to occur in elderly people, but has no distinctive clinical features, including radiological features. Standard treatment of hepatic MALT lymphoma has not been established. Since it was usually misdiagnosed before histological confirmation, surgery resection may be a good choice for both diagnosis and local therapy.
